# Novel inhibitors are cytotoxic for myeloma cells with NFkB inducing kinase-dependent activation of NFkB

**DOI:** 10.18632/oncotarget.2128

**Published:** 2014-06-23

**Authors:** Yulia N. Demchenko, Leslie A. Brents, Zhihong Li, Leif P. Bergsagel, Lawrence R. McGee, Michael W. Kuehl

**Affiliations:** ^1^ Genetics Branch, National Cancer Institute, Bethesda, MD, USA; ^2^ Amgen, Inc., South San Francisco CA, USA; ^3^ Comprehensive Cancer Center, Mayo Clinic Arizona, Scottsdale

**Keywords:** NFkB-inducing kinase, NIK inhibitors, NFkB, IKKbeta inhibitors, multiple myeloma.

## Abstract

NFkB activity is critical for survival and proliferation of normal lymphoid cells and many kinds of B-cell tumors, including multiple myeloma (MM). NFkB activating mutations, which are apparent progression events, enable MM tumors to become less dependent on bone marrow signals that activate NFkB. Mutations that activate NFkB-inducing kinase (NIK) protein are the most prevalent among the many kinds of NFkB mutations in MM tumors. NIK is the main activating kinase of the alternative NFkB pathway, although over-expression of NIK also can activate the classical pathway. Two NIK inhibitors and an isomeric control were tested with human myeloma cell lines. These specific NIK inhibitors are selectively cytotoxic for cells with NIK-dependent activation of NFkB. Combination therapy targeting NIK and IKKbeta (as a main kinase of the classical NFkB pathway) represents a promising treatment strategy in MM. NIK inhibitors can also be useful tool for assessing the role of NIK and alternative NFkB pathway in different cells.

## INTRODUCTION

Multiple myeloma (MM) is a mostly incurable, age dependent monoclonal tumor of long-lived bone marrow plasma cells (PC), usually with significant end organ damage[1]. It is the second most common hematopoietic malignancy, with an incidence of about 20,000 per year in the United States[2]. The median survival is about five years, thus accounting for nearly 2% of deaths from cancer. Often MM is preceded by a pre-malignant tumor called monoclonal gammopathy of undetermined significance (MGUS), with a prevalence of about 3% of individuals over the age of 50[3, 4]. MGUS cells are similar to post-germinal center long-lived PC, including strong BM dependence, but differ from long-lived PC by retaining the potential for a low rate of proliferation (1-3% of cycling cells)[5].

NFkB transcription factors play a key role in the survival and/or proliferation of normal PC, and also of MGUS and MM tumors[6-8]. The NFkB family of transcription factors is composed of 5 subunits - NFKB1 (p50 and its precursor p105), NFKB2 (p52 and its precursor p100), RelA (p65), RelB and c-Rel[9, 10]. The classical NFkB pathway can be activated by different stimuli, including external signaling through B cell receptors (BCR) and some tumor necrosis factor receptors (TNFR). Activation of IKKβ (which is part of an IKKα-IKKβ-IKKγ complex) by signals from TNFR-associated factors, leads to phosphorylation and proteasomal degradation of the inhibitory subunits IkBα, IkBβ, and IkBε. As a result, NFkB homodimers and heterodimers, comprised mainly of RelA, RelC, and p50, accumulate in the nucleus. Classical RelA:p50 heterodimers are predominantly regulated by IkBα. This pathway participates in various biological processes, including immune response and inflammation, and is required to enhance the survival and proliferation of cells.

The alternative NFkB pathway is important in lymphoid development and B-cell maturation[11]. One of the critical events in the alternative NFkB pathway is accumulation of NFkB-inducing kinase (NIK), which phosphorylates IKKα and NFKB2. This results in proteasomal removal of an inhibitory C-terminal IkBδ domain, generating the p52 subunit, which leads to accumulation of p52/RelB heterodimers in the nucleus. Several cytokines, including CD40L, LTαβ, BAFF, RANKL (receptor activator of NFkB ligand), and TWEAK (TNF-related weak inducer of apoptosis)[12-14] can activate the alternative pathway, mainly through the control of NIK turnover. Under normal conditions, the level of NIK protein is extremely low, due to its constant degradation through an ubiquitination-dependent mechanism mediated by a negative regulatory complex, composed of TRAF3, TRAF2 and cIAP1/2 proteins[15-18]. TRAF2/TRAF3 interaction recruits a TRAF2-cIAP1/2 ubiquitin ligase complex to a TRAF3-NIK complex, which results in the cIAP1/2-mediated K48 ubiquitination of NIK that marks it for rapid proteasomal degradation. It was shown that high levels of NIK protein activate the alternative pathway but also the classical pathway in most MM cell lines [15-18].

Extrinsic ligands (APRIL and BAFF) produced by BM stromal cells provide critical survival signals to long-lived PC by stimulating TACI, BCMA, and BAFF receptors to activate the NFkB pathways[19]. Similar to PC, most MGUS and MM tumors highly express NFkB target genes, suggesting a continued important role of extrinsic NFkB ligands in PC tumors[20, 21]. However, mutations occurring during MM progression can constitutively activate the classical and/or alternative NFkB pathways, with consequent increased tumor autonomy. Activating mutations in six positive regulators and inactivating mutations in five negative regulators of the NFKB pathway have been identified in 20% of untreated MM tumors and 45% of multiple myeloma cell lines (MMCLs), rendering the cells less dependent on ligand mediated NFKB activation. In addition whole genome sequencing studies identified apparent NFKB activating mutations in 11 (29%) of 38 MM tumors, including identification of mutations in an additional 8 genes[22]. Mutations that result in increased levels of NIK protein are overrepresented, with a substantial prevalence of TRAF3 mutations in both MMCLs (one-third of mutations) and MM tumors.

Given the strong dependence of MM tumors on extrinsic and/or intrinsic NFkB pathway activation, and the frequent activation of both NFkB pathways, simultaneous inhibition of both NFkB pathways might be an attractive therapeutic strategy for the treatment of MM tumors. Small molecules that inhibit extrinsic signaling (TACI.Fc) or intrinsic IKKβ are being developed as potential therapeutic agents[23-25]. NIK is the key regulator of the alternative pathway, but the number of specific inhibitors of the alternative NFkB pathway is limited [26, 27].

In our studies we tested three new Amgen compounds (two NIK inhibitors [AM-0216, AM-0561] and an enantomeric control [AM-0650]) with different multiple myeloma cell lines, and showed high specificity and selectivity of these new NIK inhibitors[28].

## RESULTS

### NIK inhibitors are specific for MMCLs with NIK-dependent NFkB activation

**Figure 1:**
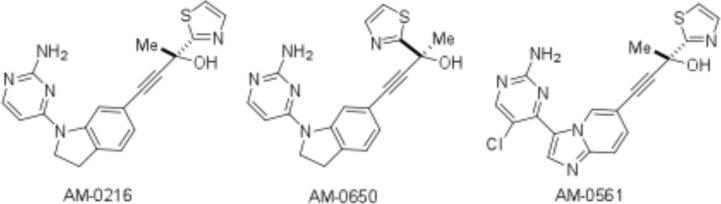
The structure of Amgen NIK compounds

Two NIK inhibitors [AM-0216, AM-0561] and an isomeric control of AM-0216 [AM-0650] were used in our experiments (Fig. 1). AM-0216 inhibits NIK with a Ki of 2 nM in an HTRF assay[28]. AM-0650 is the enantiomer of AM-0216, with a Ki of 290 nM against NIK, consistent with >99% optical purity. AM-0561 is a more potent active analog with a Ki of 0.3 nM. The KINOMEscan ^®^ survey and K_d_ELECT assays were done for Amgen (Table S1-S4) by DiscoveRx. Details about these competition binding assays have been published[29, 30] and can be found on the Discoverx website [http://www.discoverx.com/services/drug-discovery-development-services/kinase-profiling/kinomescan].

**Figure 2:**
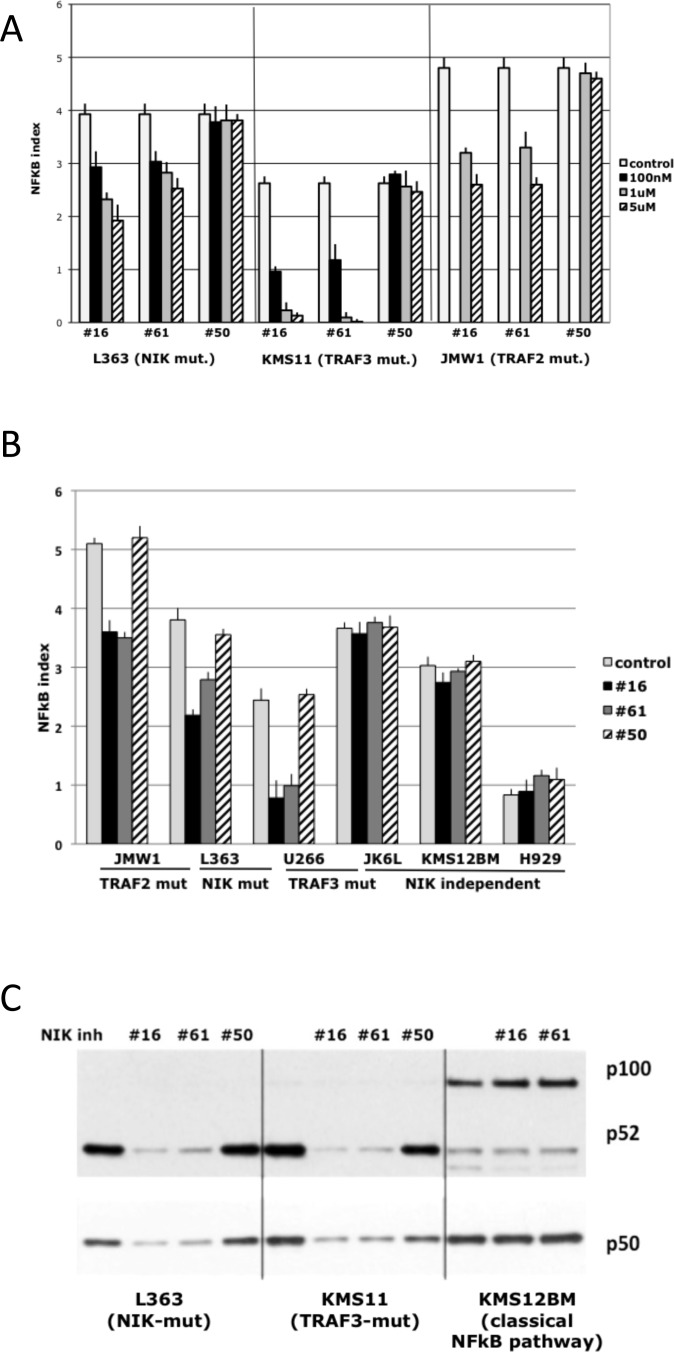
Effect of NIK inhibitors on NF-kB activity in MM cells with different genetic abnormalities of NF-kB pathway components (A) NFkB index in MM cell lines treated with NIK compounds* for 16 h. The NFkB index is the average expression (log2) of 3 target-genes (cIAP2, TNFAIP3, NFKB2) as determined by qRT-PCR. (Data are mean ±SD of triplicate experiments and significance determine by a t-test). (For details see “Materials and Methods”). (B) NFkB target gene expression following inhibition of NIK activity in MM cell lines after 16 h of incubation with 1 uM NIK inhibitors. (Data are mean ±SD of triplicate experiments).(C) Immunoblot of nuclear extracts of myeloma cell lines cultured for 16 h in medium in the presence of 0.1% DMSO or 1 uM NIK-inhibitors. Nuclear extracts were prepared, and expression of NFKB1 (p50) and NFKB2 (p100 and p52) was analyzed. *- AM-0216 (#16), AM-0561 (#61), AM-0650 (#50).

Amgen compounds were tested on MMCLs that have mutations (NIK, TRAF2, TRAF3, CIAP1&2) leading to the activation of NIK. As a control, these compounds were tested on MMCLs with mutations that activated either the classical NFkB or alternative pathway by NIK-independent mechanisms, and also in MMCLs without NFkB related mutations, which have low NFkB activity. MMCLs were treated with different concentrations of inhibitors or control compound for 16 h, and then NFkB activity was measured by determining the RNA expression levels of 3 NFkB target genes - cIAP2, TNFAIP3, NFKB2, which are the most sensitive target genes in myeloma cells [15]. Dose-dependent NFkB inhibition was demonstrated in three NIK-dependent cell lines: L363 (with a translocation that increases NIK expression), KMS11 (with homozygous inactivation of the TRAF3 gene), and JMW1 (with homozygous inactivation of the TRAF2 gene)[15, 20] (Fig. 2A). The most effective inhibitor concentrations in cell-based assays were 1-5 uM. Concentrations higher then 5 uM cause nonspecific toxicity, as evidenced by the toxicity induced by compound AM-0650 at these higher concentrations (data not shown). Comparison of NFkB activity in different MMCLs after treatment with NIK-inhibitors showed that compounds AM-0216 and AM-0561, but not AM-0650, decrease NFkB activity only in cells in which NFkB activity is NIK-dependent (Fig. 2B). The efficiency of inhibitors was variable for different MMCLs, consistent with decreased sensitivity related to higher NIK protein levels and a higher NFkB index. For example, JMW1 cells (TRAF2 mutation), which have one of the highest levels of NIK protein and NFkB index, were less sensitive to inhibitors than U266 cells (TRAF3 mutation), which had much lower levels of NIK protein and NFkB index. The specificity of NIK-inhibitors was also confirmed by a Western blot (Fig. 2C), which showed that both NIK-inhibitors, but not the control compound, decrease the level of nuclear p52 and p50 proteins in NIK-dependent MMCL (as the overexpressed NIK can activate not only the alternative but also the classical pathway). Together, these data confirm that NIK-inhibitors block both classical and alternative NFkB pathways in a dose-dependent manner in MMCLs that have NIK-dependent activation of NFkB.

### NIK-inhibitors can effectively block NFkB activity induced by smac-mimetic

**Figure 3:**
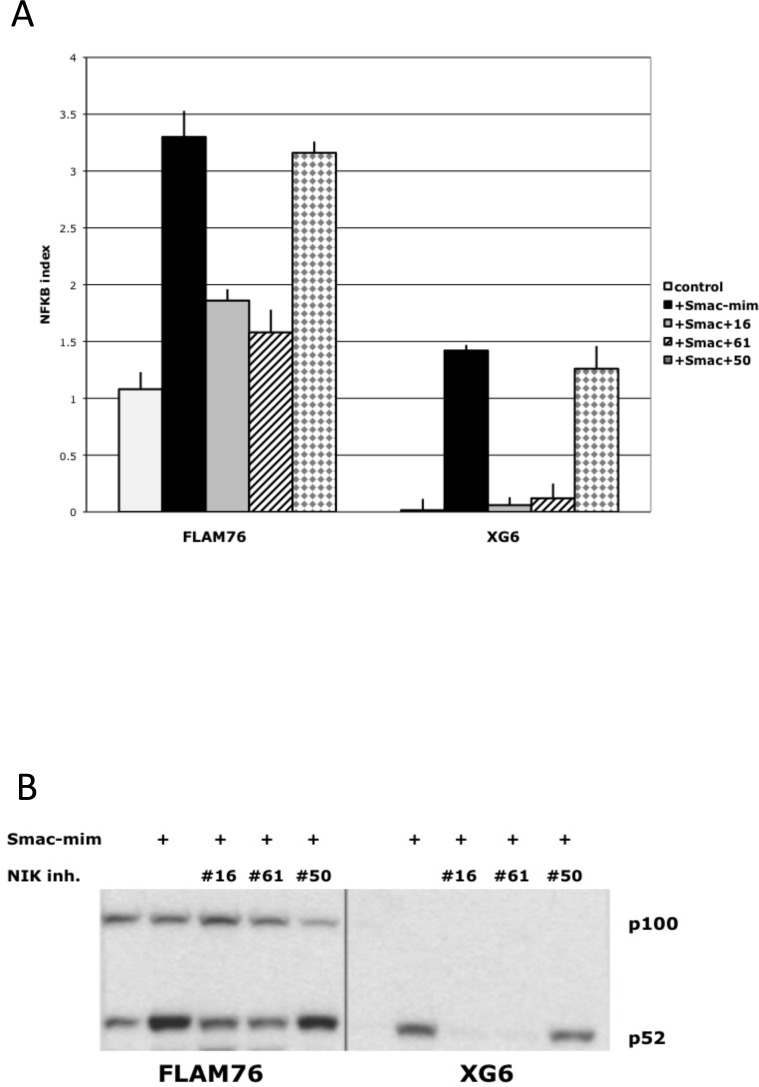
NIK compounds* inhibit NFkB activation mediated by Smac-mimetic in MM cells (A) NFkB target gene expression following 16h treatment of MM cells by 0.1% DMSO (control), or 50 nM Smac-mimetic with or without 1 uM NIK-inhibitors. The NFkB index is the average expression (log2) of 3 target-genes (cIAP2, TNFAIP3, NFKB2) as determined by qRT-PCR. (Data are mean ±SD of triplicate experiments and significance determine by a t-test). Immunoblot of nuclear extracts of myeloma cell lines cultured for 16 h in medium in the presence of 0.1% DMSO or 50 nM Smac-mimetic with or without 1 uM NIK-inhibitors. Nuclear extracts were prepared, and expression of NFKB1 (p50) and NFKB2 (p100 and p52) was analyzed. *- AM-0216 (#16), AM-0561 (#61), AM-0650 (#50).

It is not possible to measure the efficiency of inhibitors on MMCLs with high NFkB activity because we don't know the basal level of the NFkB index in the absence of elevated NIK protein levels. Therefore, several MMCLs (XG-6, FLAM-76) without mutations in NFkB pathway and with a low basal NFkB index were treated with a smac-mimetic that causes increased degradation of XIAP and CIAP1/2,[31, 32] which results in increased levels of NIK protein and increased NFkB activity (Fig. 3A). Treatment of these cells with NIK inhibitors markedly inhibited the smac-mimetic induced increase in NFkB activity (~80-90% inhibition for FLAM76 cells and >95% for XG6 cells). Western blot analysis confirmed these results (Fig. 3B) by showing that NIK inhibitors AM-0216 and AM-0561 (but not control AM-0650) restore nuclear p52 to the nearly the same level present before smac-mimetic treatment. Taken together, our results indicate that NIK-inhibitors have high specificity, and can in some cases almost completely block NIK-dependent NFkB activity.

### NIK-inhibitors induce cytotoxicity and apoptosis in MM cells

**Figure 4:**
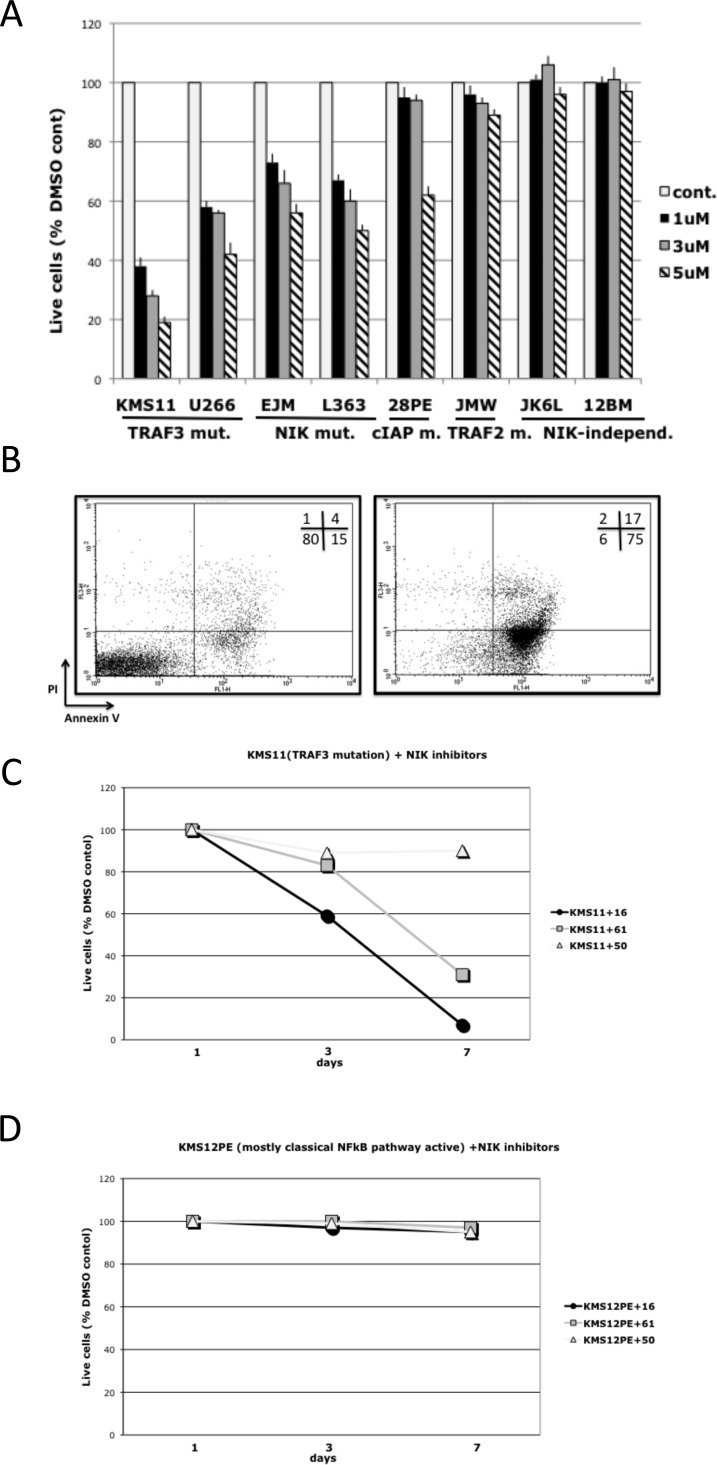
NIK inhibitors* can selectively decrease viability of MMCLs in which NFkB activity is NIK-dependent (A) MMCLs were cultured in the presence of NIK inhibitor AM-0561. After 7 days, cell viability was determined by CellTiter-Glo luminescent cell viability assay, and displayed relative to a control culture treated with the same volume of DMSO. (Data are mean ±SD of triplicate experiments and significance determined by a t-test). (B) Representative FACS analysis of KMS11 cells after treatment for 7 days with DMSO control or 1 uM compound AM-0216, stained with AnnexinV-PI kit. Cell lines with NIK-dependent NFkB activation: (C) KMS11 (TRAF3 mutation) or NIK-independent NFkB activation: (D) KMS12BM (classical NFkB pathway activation) were cultured in the presence of 1 uM NIK inhibitors. After 3 and 7 days, cell viability was determined by flow cytometry with the Annexin-V-FLUOS Staining Kit, and displayed relative to a control culture treated with the same amount of DMSO. * – AM-0216 (#16), AM-0561 (#61), AM-0650 (#50).

Treatment of MMCLs that have NIK-dependent activation of NFkB with inhibitors for 7 days induced a dose-dependent decrease in cell viability, but these inhibitors did not decrease viability of cells that have NIK-independent activation of NFkB (Fig. 4A). Greater sensitivity to the NIK inhibitors was observed in MMCLs with TRAF3 mutations, whereas MMCLs with TRAF2 and CIAP1/2 mutations were less sensitive, consistent with previous findings that the increased level of NIK is generally related to the type of mutation, e.g., TRAF2 ~ CIAP1/2 > TRAF3[15]. To confirm that NIK-inhibitors induce cytotoxicity in MMCL cells, L363, KMS11, JK6L and KMS12BM cells treated with NIK-inhibitors were analyzed for apoptosis using AnnexinV-PI staining. NIK-inhibitors significantly increased the number of AnnexinV(+)/PI(-) and AnnexinV(+)/PI(+) cells in a time–dependent manner only for the two MMCL with NIK-dependent activation of NFkB (Fig. 4B-D, Fig. S1A-B). Taken together, these results show that NIK–inhibitors, in a dose- and time-dependent manner, induced apoptosis in MMCLs that have mutations leading to the activation of NIK.

### NIK-inhibitors prevent generation of clones from NIK-dependent MMCLs

**Figure 5:**
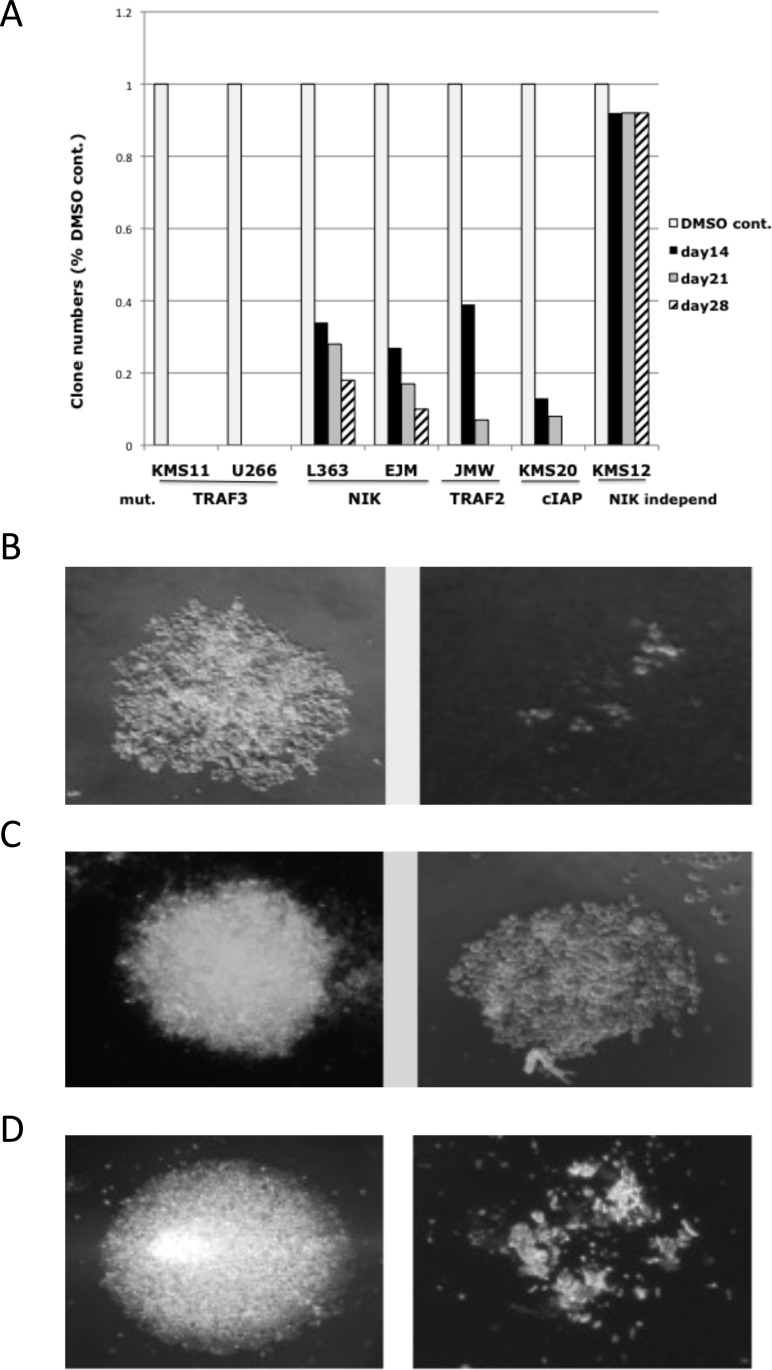
Effect of NIK inhibitor on MMCLs in a limiting dilution cloning assay (A) Number of clones of MMCLs with different NFkB mutations in the presence of 1 uM NIK-inhibitor (AM-0561(#61)) compared to DMSO-control. (B) JMW MMCL DMSO-control (left) and cells treated with 1 uM NIK-inhibitor (right) after 28 days. (C) Clones generated from the EJM cell line in the present of DMSO or 1 uM NIK-inhibitor after 28 days (note smaller size of clone). (D) The clones generated from EJM in 1 uM NIK inhibitor, were divided into three aliquots. One aliquot was treated with DMSO, and the other two aliquots with 1 uM (not shown) and 3 uM NIK-inhibitor. On photo – DMSO control and cells with 3 uM compound AM-0561 after 28 days.

The experiments above showed that the NIK inhibitors decreased viability of cell lines with NIK-dependent NFkB activity, but the extent of inhibition could not be fully assessed in short term experiments on bulk cultures. Therefore, we used a limiting dilution cloning assay to show that all NIK-dependent cell lines tested were extremely sensitive to NIK inhibitors, including MMCLs with overexpression of NIK RNA or inactivation of TRAF2, TRAF3 or CIAP1/2 (Fig. 5A). By contrast, the NIK inhibitors do not significantly affect the cloning efficiency of the KMS-12BM MMCL with NIK-independent activation of NFkB. Some clones were formed for MMCLs with the highest levels of NIK protein (L363, EJM, JMW, KMS-20) but the size of these clones was much smaller than in control (Fig. 5B,C). Three of the smaller clones generated from the EJM MMCL in the presence of 1 uM NIK inhibitor (AM-0261) were isolated and divided into three aliquots. In each case, one aliquot was re-cultured with the solvent control, and the other two aliquots with 1 or 3 uM NIK-inhibitor. In the absence of the NIK inhibitor, all three of the smaller clones grew at a rate similar to the original cells cultured in the absence of inhibitor. However, in the presence of 1 uM inhibitor, the cells from one clone failed to grow, whereas the cells from the other two clones continued to grow slowly. However, cells from all three clones failed to grow with 3 uM NIK inhibitor (Fig. 5D). These results confirmed that all NIK-dependent MMCLs are sensitive to NIK inhibitors, but that some MMCLs require higher inhibitor concentration for complete inhibition.

### NIK-independent activation of NFkB pathway can minimize the cytotoxic effect of NIK-inhibitors

To further confirm the specificity of NIK-inhibitors, we transfected NIK-dependent KMS-11 cells with constitutively active IKKβ that activates the classical NFkB pathway, or NFKB2 (p52) that activates the alternative pathway independently of NIK. The NFkB indices in the cells transfected with IKKβ and with p52 were higher than in cells transfected with empty vector (Fig. S2A). Treatment of these transfected populations with 2 uM NIK inhibitor (AM-0216) for 16 hours decreased the NFkB indices for control KMS11 cells by 82%, but only by 22% and 7% in cells transfected with p52 and IKKβ, respectively (Fig. S2A). Incubation of these cells during 7 days with the 2uM NIK-inhibitors showed that the NIK-independent activation of either the classical or alternative NFkB pathways could substantially decrease the cytotoxic effect of inhibitors (Fig. S2B).

### IKKβ inhibitors and dexamethasone enhance cytotoxicity of NIK-inhibitors in MMCLs with NIK-dependent NFkB activation

Previously, it was shown that most MM cell lines with high NFkB activity are sensitive to IKKβ inhibitors [20, 21]. We evaluated the combination of NIK-inhibitors with a small molecule IKKβ inhibitor (MLX) in L363 (with a translocation that increases NIK expression) and KMS11 (with homozygous inactivation of the TRAF3 gene) MM cell lines. Treatment of these cells with or without 25 uM MLX and 1 uM of all 3 compounds (AM-0216, AM-0561 and AM-060) showed that combination of AM-0216 or AM-0561 with MLX decreased NFkB activity more effectively than either of these compounds alone (Fig. S3A, B). Similar results were obtained also with combination of NIK-inhibitors and dexamethasone (Fig. S3C). These data confirm that NIK-inhibitors in combination with other cytotoxic agents much more effective decrease the viability of MMCLs.

### NFkB activation induced by BAFF is fully inhibited by a combination of a NIK-inhibitor and an IKKβ inhibitor

**Figure 6:**
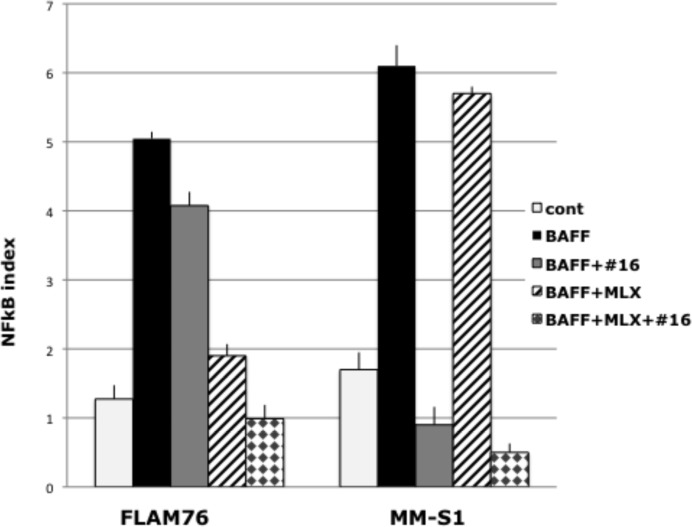
The combination of NIK and IKKβ inhibitors can block BAFF induced NFkB activation in myeloma cells NFkB target gene expression in FLAM76 and MMM1 cell lines after 16 h of incubation with or without 200ng BAFF, 1 uM of NIK inhibitor AM-0216 (#16) or 25 uM of MLX. The NFkB index is the average expression (log2) of 3 target-genes (cIAP2, TNFAIP3, NFKB2) as determined by qRT-PCR (Data are mean ±SD of triplicate experiments and significance determined by a t-test).

The interaction of BAFF with BAFFR receptors can increase NIK levels, with consequent NIK-dependent activation of both the alternative and classical NFkB pathways. Alternatively, BAFF can interact with BCMA or TACI receptors to cause NIK-independent activation of the classical NFkB pathway. FLAM 76 and MM-S1 are MMCL without known mutations in the NFkB pathway and with low NFkB indices. Treatment of these MMCLs with BAFF markedly increased the NFkB indices (Fig. 6), as well as the levels of p52 and p50 nuclear protein (not shown). For both MMCLs, the combination of a NIK-inhibitor and MLX, an IKKβ inhibitor, fully blocked the BAFF-induced NFkB activation. For the FLAM76 MMCL, there was minimal inhibition by the NIK-inhibitor but marked inhibition by the IKKβ inhibitor, suggesting that the BAFF activation was mediated primarily by its interaction with BCMA and/or TACI receptors. The opposite result was obtained for the MM-S1 MMCL, suggesting that the BAFF activation was mediated primarily by its interaction with BAFFR receptors.

### Stromal cells do not negate the cytotoxic effect of NIK inhibitors on a primary MM tumor or the corresponding MMCL that have inactivated TRAF3

**Figure 7:**
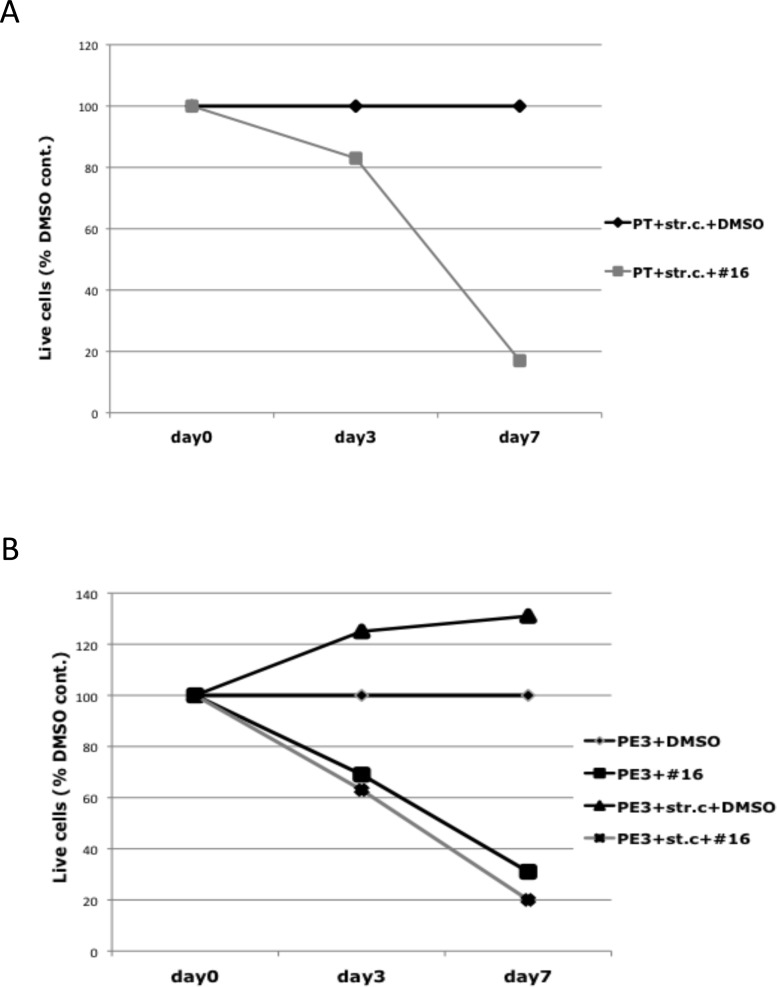
Effect of NIK inhibitor on myeloma cells in the presence of stromal cells (A) MC1286 primary tumor cells (PT) or (B) MC1286.PE3 cells (PE3) were co-cultured with BMSC in the presence or absence of 2 uM of NIK inhibitor AM-0216 (#16). After 7 days, cell viability was determined by cell viability analysis using Trypan Blue staining, and displayed relative to a control culture treated with the same volume of DMSO.

Purified primary MC1286 tumor cells that had a homozygous deletion of TRAF3 were purified, and then co-cultured with primary bone marrow stromal cells for 7 days, with or without inclusion of 2 uM NIK inhibitor AM-0216. The number of live cells remained at a constant level for 7 days in the absence of inhibitor, but decreased by more than 80% in the presence of inhibitor (Fig. 7A). A TRAF3 deficient MC1286.PE3 MMCL was generated from primary myeloma MC1286 cells during a subsequent relapse. Bone marrow stromal cells stimulated proliferation of the MC1286.PE3 MM MMCL, which did not proliferate when cultured without IL-6 or stromal cells (Fig. 7B). Nonetheless, the TRAF3 deficient MMCL showed a similar sensitivity to the NIK inhibitor, with or without the inclusion of bone marrow stromal cells.

## DISCUSSION

The treatment of MM has significantly improved over the past decade, mainly due to recently approved drugs, such as bortezomib, thalidomide and lenalidomide[33-35]. Increasing knowledge of the important role of the NFkB pathway in MM extend the potential for the development of novel drugs that target this pathway.

In principle, the NFkB pathway could be targeted in MM tumors by blocking extrinsic activation of the pathway mediated by factors produced in the BM microenvironment, or by directly targeting intrinsic components of this pathway. The sequestration of BAFF and APRIL ligands by TACI.Fc results in a substantial decrease of NFkB activity in normal murine BM PC and MM tumors[8, 36, 37]. An initial clinical trial treating MM patients with Atacicept (TACI.Ig) showed that the treatment is well-tolerated and decreased the amount of tumor in some patients[23, 24]. To enhance the inhibition of extrinsic signaling, and especially for the significant fraction of tumors that have mutations in the NFkB pathway, it would seem useful to simultaneously include agents that block intrinsic components of both the classical and alternative NFkB pathways. In fact, several inhibitors of IKKβ kinase, a critical component of the classical NFkB pathway have been developed. It has been shown that some of the IKKβ kinase inhibitors efficiently prevented growth of myeloma cells and induced apoptosis through caspase activation[20, 25].

In view of findings that a substantial shRNA knock-down of IKKα kinase has no effect on NFkB activity in MMCL[20], NIK protein would seem to be a better target for drug development for the alternative pathway for the following reasons. First, NIK is the key regulator of the alternative pathway and is the ultimate target of most of the mutations affecting the NFkB pathway in MM[20, 21]. Second, a knock-down of NIK expression with anti-NIK shRNA can inhibit both the alternative and classical NFkB pathways when both are activated as a result of increased levels of NIK[15, 20], but the number of specific inhibitors of the alternative NFkB pathway is extremely limited [26].

Our results showed that two NIK inhibitors (but not an isomeric control), at concentrations of 1-5 uM, are selectively toxic for MMCLs that have different kinds of mutations resulting in NIK-dependent activation of NFkB. Importantly both inhibitors had no effect on NFkB activity or cell viability of MMCLs that had little or no activation of the NFkB, or on MMCLs that had mutations selectively activating the classical NFkB pathway. However, if MMCLs with little or no NFkB activity were treated with a smac-mimetic to induce increased NIK protein levels and NFkB activity, the NIK inhibitors almost completely blocked the increased NFkB activity. It is well established that increased expression of NIK protein can activate both the alternative and classical NFkB pathways in MMCLs. Consistent with a specific effect of these two inhibitors on NIK, we showed that they caused a decrease in the level of nuclear subunits that mediate both the alternative (p52) and classical (p50) NFkB pathways. Although p52 is localized in the nucleus and cytoplasm, p100 usually is localized mostly in the cytoplasm. Therefore it was not unexpected that the NIK inhibitors caused a decreased level of nuclear p52 but not a corresponding increased level of nuclear p100. However, as shown previously[15], for some MMCL there is a significant concentration of nuclear p100 (see also Figs. 2C and 3B). We cannot explain this finding but note that this occurs mostly with MMCL that do not have activation of the alternative NFkB pathway, with consequent processing of p100 to p52. MMCLs with the highest levels of NIK protein (e.g. CIAP1/2 or TRAF2 mutations) were somewhat less sensitive to the NIK inhibitors than MMCLs with lower levels of NIK protein (e.g., TRAF3 mutations). Nonetheless, we used a limiting dilution cloning assay to show that 1-3 uM NIK inhibitor markedly blocked the growth of all MMCLs tested that had NIK-dependent activation of NFkB.

Therefore these NIK inhibitors can be useful for assessing the role of NIK and also for studies of the role of two NFkB pathways in different cell lines. Thus, in our experiments we showed that replacement of NIK activity with constitutive active IKKβ or p52 can minimize the toxic effect of NIK inhibitors in KMS11 myeloma cells. This supports our previous conclusion that activation of either NFkB pathways has a similar effect in myeloma cells [15]. It is sometimes quite difficult to define the main pathway of NFkB activation because of strong crosstalk between these two pathways, as in our experiments with BAFF, when we saw activation of both pathways. But our experiments with NIK inhibitors indicate that in the MM.S1 MMCL this activation was mostly NIK-dependent whereas in the FLAM76 MMCL it mostly was NIK-independent.

However, despite the specificity of these inhibitors for NIK, it was not possible to do animal experiments because of the poor *in vivo* pharmacokinetic properties of these inhibitors. But coculture of MC1286.PE3 cell line and primary tumor sample with BMSCs gave promising results and showed that modified Amgen NIK inhibitors could be effective for *in vivo* studies. The combination of those NIK inhibitors and IKKβ-inhibitors or dexamethasone may also provide an effective therapeutic strategy to more efficiently target most MM tumors, including tumors that do not have intrinsic mutations in the NFkB pathway, but are dependent on extrinsic ligands that activate the NFkB pathway.

## MATERIALS AND METHODS

### Cell Culture and Transfections

MMCLs were maintained in RPMI 1640 or Advanced RPMI medium supplemented with fetal calf serum (Hyclone) and penicillin/streptomycin (Invitrogen), with or without 10 ng/ml IL-6 (R&D Systems). Bone marrow stromal cells, MC1286.PE3 cell line and primary MC1286 tumor cells were kindly provided by P. Leif Bergsagel (Comprehensive Cancer Center, Mayo Clinic Arizona, Scottsdale). Smac-mimetic was kindly provided by X. Wang (UT Southwestern Medical Center)[32]. Constitutive active IKKβ construct was kindly provided by Louis M. Staudt (NIH, NCI)[38]. Constitutive active p52 construct pBABE/p52 (codon 1-446) was kindly provided by Sivakumar Vallabhapurapu (The Vontz Center for Molecular Studies, Department of Cancer and Cell Biology, University of Cincinnati College of Medicine, Cincinnati, OH). Transductions were performed by spin infection in the presence of 8 ng/ml polybrene (Sigma) as described previously[39]. After 24 h the virus-containing medium was replaced with selection medium containing 2 ng/ml puromycin. When cell growth was stable, the cells were used in the experiments described.

### NIK Inhibitors

The NIK inhibitors are described in WO 2009158011 A1[28].

AM-0216 is example 294 (R)-4-(1-(2-aminopyrimidin-4-yl)indolin-6-yl)-2-(thiazol-2-yl)but-3-yn-2-ol. AM-0561 is example 296 (R)-4-(3-(2-amino-5-chloropyrimidin-4-yl)imidazo[1,2-a]pyridin-6-yl)-2-(thiazol-2-yl)but-3-yn-2-ol. AM-0650 (the enantiomer of AM-0216) is example 297 (S)-4-(1-(2-aminopyrimidin-4-yl)indolin-6-yl)-2-(thiazol-2-yl)but-3-yn-2-ol. Details of the synthesis and activity of these molecules will be published elsewhere (manuscript in preparation). Compounds were added to cultures in a required volume from 1 mM DMSO stock solution, with controls having the same final concentration of DMSO.

### Western Blot

Protein was harvested from MMCLs, and fractionated using a Nuclear/Cytosol fractionation kit (BioVision). Purity of the nuclear fraction was checked with b-tubulin antibodies (not shown). Protein was quantified using the BCA method (Pierce), and separated by SDS-PAGE on a 4%–12% acrylamide gradient. The following antibodies were used: p50/p105 (Cell signaling), p52/p100 (Upstate), B-tubulin (Sigma).

### qRT-PCR

Total RNA from cells was isolated using the TRIZOL reagent (Gibco BRL, Rockville, MD). First-strand complementary DNA (cDNA) synthesis was performed by using High Capacity cDNA RT Kit (Applied Biosystems, Foster City, CA). The following TaqMan probes: Hs00231528_m1 USF2, Hs00985031_g1 BIRC3 (aka cIAP2), Hs00234712_m1 TNFAIP3, Hs00174517_m1 NFKB2 and the TaqMan Fast Universal PCR Master Mix Reagents kit (Applied Biosystems) were used. The comparative C_T_ method (ΔC_T_) was used for relative quantification of gene expression (C_T_ of target genes minus C_T_ of reference USF2 gene). An NFkB index was determined as the average expression (log2) of 3 target genes (cIAP2, TNFAIP3, NFKB2).

### Cell Viability Assays

MMCLs were seeded onto 96-well plates at a density of ~5×10^4^ cells/well in a volume of 0.2 ml of media. After 7 days of incubation in the presence or absence of 1-5 uM NIK-inhibitor (AM-0216 or AM-0561) or DMSO, cells were analyzed for cell viability by the addition of CellTiter Glo (Promega, Madison, WI) to the assay plates. The signal from the viable cells was analyzed on a Victor X4 (PerkinElmer).

### Flow Cytometric Studies

For apoptosis/necrosis detection MMCLs were treated with compounds (1uM NIK inhibitors, 25 uM MLX or 20 uM Dexamethasone) or DMSO for 3 or 7 days. The cells were washed and resuspended in Annexin-V/propidium iodide buffer solution according Annexin-V-FLUOS staining kit (Roche) protocol. Samples were immediately analyzed on a FACScan (Becton Dickinson).

### Cloning by limiting dilution

One or three cells/well were plated in 0.2 ml aliquots in 96 wells of TPP (Switzerland) round bottom plates for each cell density. The plates were incubated for 28 days in the presence or absence of 1 uM NIK-inhibitor (AM-0216 or AM-0561) or DMSO, without subsequent changes to the medium, and observed for growth after 14, 21 and 28 days. The cloning efficiencies in the DMSO controls were 30-60% for the MMCLs tested.

## SUPPLEMENTARY TABLES AND FIGURES


