# Supplementary material for: Novel inhibitors are cytotoxic for myeloma cells with NFkB inducing kinase-dependent activation of NFkB
Source: Oncotarget. 2014 Jun 23;5(12):4554–66. doi: 10.18632/oncotarget.2128 (PMC4147345; doi:10.18632/oncotarget.2128)
Supplement: Supplementary file 1 [file oncotarget-05-4554-s001.pdf]

Novel inhibitors are cytotoxic for myeloma cells with NFkB inducing kinase-dependent activation of NFkB

Supplementary Material

Table S1: Summary of the KINOMEscan® survey:

Data set 1

Compound Name	Selectivity Score Type	Number of Hits	Number of Non- Mutant Kinases	Screening Concentration (nM)	Selectivity Score
AM-0216#2	S(35)	8	99	1000	0.081
AM-0216#2	S(10)	5	99	1000	0.051
AM-0216#2	S(1)	2	99	1000	0.02
AM-0561#2	S(35)	7	99	1000	0.071
AM-0561#2	S(10)	1	99	1000	0.01
AM-0561#2	S(1)	1	99	1000	0.01
AM-0650#2	S(35)	6	99	1000	0.061
AM-0650#2	S(10)	2	99	1000	0.02
AM-0650#2	S(1)	1	99	1000	0.01

data set 2

Compound Name	Selectivity Score Type	Number of Hits	Number of Non- Mutant Kinases	Screening Concentration (nM)	Selectivity Score
AM-0216#2	S(35)	10	99	1000	0.101
AM-0216#2	S(10)	5	99	1000	0.051
AM-0216#2	S(1)	2	99	1000	0.02
AM-0561#2	S(35)	8	99	1000	0.081
AM-0561#2	S(10)	4	99	1000	0.04
AM-0561#2	S(1)	1	99	1000	0.01
AM-					

**0650#2**	**S(35)**	**5**	**99**	**1000**	**0.051**
**AM-0650#2**	**S(10)**	**2**	**99**	**1000**	**0.02**
**AM-0650#2**	**S(1)**	**1**	**99**	**1000**	**0.01**

Each experiment duplicated.

Details about these competition binding assays have been published(1, 2) and can be found on the http://www.discoverx.com/services/drug-discovery-development-services/kinase-profiling/kinomescan DiscoveRx website.

Table S2: The actual POC values for the kinases that show up in the various S() values.

	AM-0216#2	AM-0216#2	AM-0650#2	AM-0650#2	AM-0561#2	AM-0561#2
CSNK1D	84	36	100	77	31	16
CSNK1G2	52	29	100	96	90	69
CSNK2A1	94	80	90	77	13	4.5
HIPK1	82	98	32	100	63	70
JNK3	99	100	86	100	29	22
MAP4K4	1.4	2.6	30	32	36	43
MEK3	70	57	80	74	52	27
MEK5	15	20	7.4	9.1	14	15
PRKD2	1.9	2.8	14	20	55	56
PRKG1	28	42	83	100	100	100
ROCK2	4.3	3.5	61	61	86	100
TAK1	12	11	85	58	11	8.7
TNIK	0.7	0.4	17	10	10	9.7
TSSK1B	96	27	100	80	79	37
TYK2(JH1)	46	31	99	95	84	47
YSK4	0	0	0.2	0.2	0	0
		S(35)				
		S(10)				
		S(1)				

Table S3: K_i and K_d ELECT of comparison kinases

Compound\nM	NIK Ki*	MAP4K4	PRKD2	ROCK2	TAK1	TNIK	YSK4
AMG-0216	2.1	14	34	110	39	17	0.2
AMG-0650	290	150	100	1100	1400	170	10
AMG-0561	0.33	89	610	>8000	25	120	0.9

DiscoveRx Kd Elect

*NIK is not part of DiscoveRx panel(3)

Table S4: Targets covered in KINOMEscan (NIK is not part of this panel)

KINOMEscan 99 Included Targets

ABL1-nonphosphorylated	GRK1	PIK3CD
ABL1-phosphorylated	GSK3B	PIM1
AKT1	HIPK1	PKAC-alpha
ALK	HPK1	PLK1
AMPK-alpha1	IGF1R	PLK4
AURKA	INSR	PRKCE
BMPR1A	IRAK4	PRKD2
BRAF	JAK2(JH1domain-catalytic)	PRKG1
BRK	JNK3	PRKR
BTK	KIT	RIPK2
CAMK2D	LCK	ROCK2
CAMK4	LIMK1	RPS6KA5(Kin.Dom.1-N-terminal)
CDK2	LYN	RSK1(Kin.Dom.1-N-terminal)
CDK4-cyclinD1	MAP3K1	S6K1
CDK8	MAP4K4	SRC
CHEK1	MAPKAPK2	STK33
CLK4	MARK1	SYK
CSNK1D	MEK3	TAK1
CSNK2A1	MEK5	TAOK2
DAPK1	MET	TBK1
DDR1	MKNK1	TGFBR1
DMPK	MLK1	TGFBR2
DYRK1A	MST2	TIE2
EGFR	MTOR	TNIK
EPHA2	NEK4	TRKA
EPHB3	NEK6	TSSK1B
ERK2	OSR1	TTK
ERK4	p38-alpha	TYK2(JH1domain-catalytic)
ERN1	p38-gamma	VEGFR2
FGFR1	PAK2	WEE1
FLT3	PCTK3	YANK2
FYN	PDGFRB	YSK4
GAK	PIK3CA	ZAK

See http://www.discoverx.com/services/drug-discovery-development-services/kinase-profiling/kinomescan for links to codes.

A

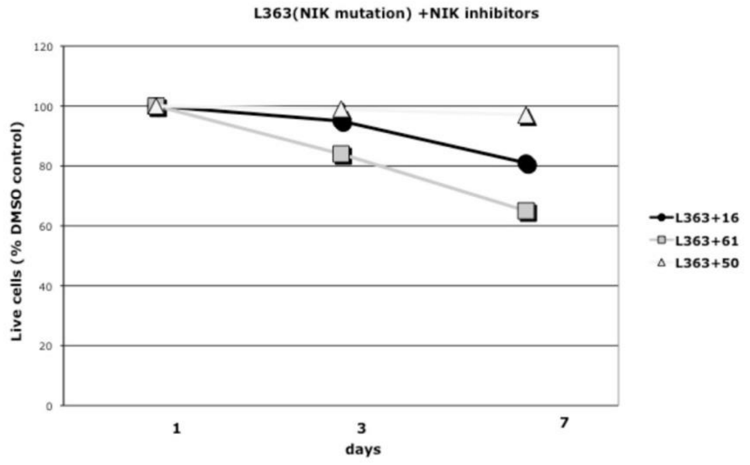

B

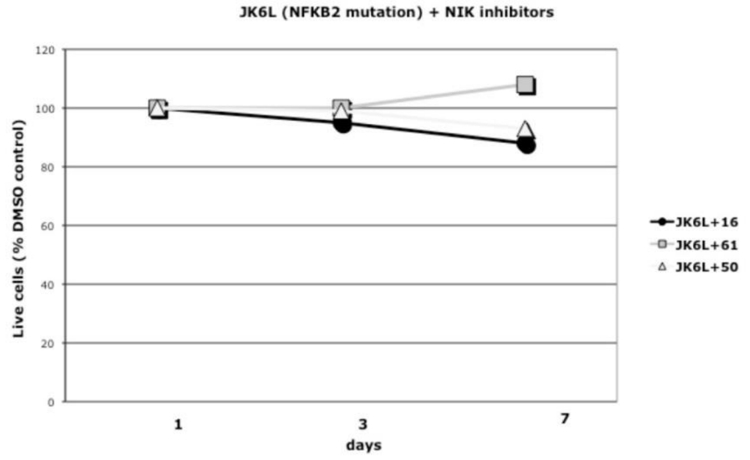

Figure S1: NIK inhibitors can selectively decrease viability of MMCLs in which NFkB activity is NIK-dependent. Cell lines with NIK-dependent NFkB activation: **(A)** L363 (NIK mutation) or NIK-independent NFkB activation: **(B)** JK6L (NFKB2 mutation) were cultured in the presence of 1 uM NIK inhibitors. After 3 and 7 days, cell viability was determined by flow cytometry with the Annexin-V-FLUOS Staining Kit, and displayed relative to a control culture treated with the same amount of DMSO

A

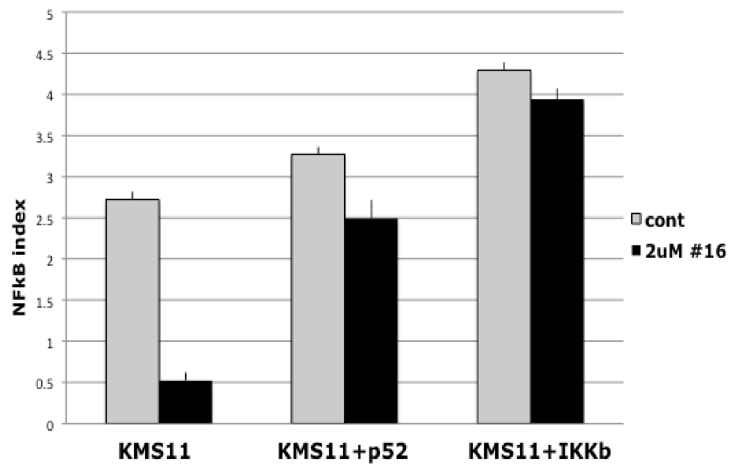

B

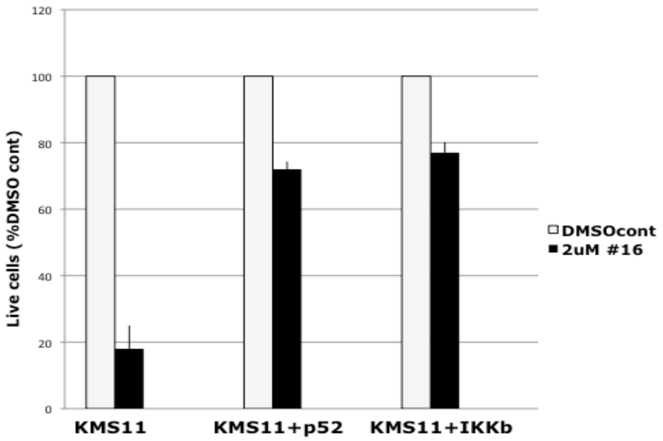

Figure S2: NIK-independent activation of NFkB pathway can minimize the cytotoxic effect of NIK inhibitor (A) NFkB target gene expression following inhibition of NIK activity in KMS11 cell line transfected with empty vector or constitutively active IKKβ or NFKB2 (p52) after 16 h of incubation with 2 uM AM-0216 (Data are mean ±SD of triplicate experiments and significance determine by a t-test). **(B)** KMS11 transfected with empty vector, constitutively active IKKβ or NFKB2 (p52) were cultured in the presence of 2 uM of NIK inhibitor AM-0216. After 7 days, cell viability was determined by cell viability analysis using Trypan Blue staining, and displayed relative to a control culture treated with the same volume of DMSO.

A

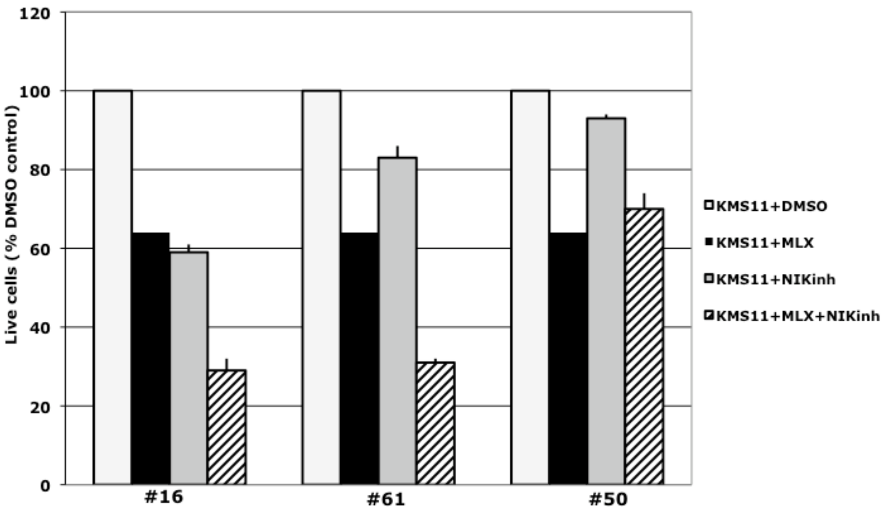
120
100
80
60
40
20
0
Live cells (% DMSO control)
#16
#61
#50
KMS11+DMSO
KMS11+MLX
KMS11+NIKinh
KMS11+MLX+NIKinh

B

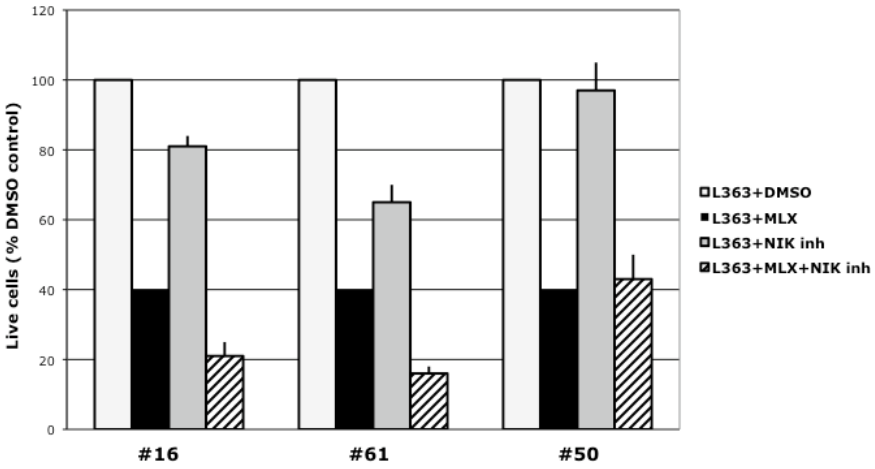
120
100
80
60
40
20
0
Live cells (% DMSO control)
#16
#61
#50
L363+DMSO
L363+MLX
L363+NIK inh
L363+MLX+NIK inh

C

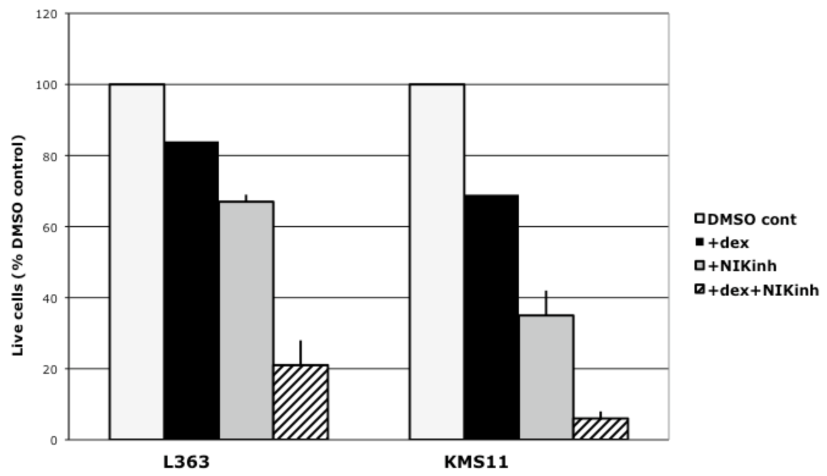

Figure S3: Effect of combination of NIK inhibitors and IKKβ inhibitor (MLX) or dexamethasone on cell viability.KMS11 and **(B)** L363 cells were cultured in the presence of 1 uM NIK compounds* and/or 25 uM MLX. After 3 days for KMS11 and 7 days for L363, cell viability was determined by flow cytometry with an Annexin-V-FLUOS Staining Kit, and the results were normalized to the DMSO solvent control. * – AM-0216 (#16), AM-0561 (#61), AM-0650 (#50). **(C**) KMS11 and L363 cells were cultured in the presence of 1 uM NIK compounds AM-0561 and/or 20 uM Dexamethasone. After 7 days cell viability was determined by flow cytometry with an Annexin-V-FLUOS Staining Kit, and the results were normalized to the DMSO solvent control (Data are mean ±SD of triplicate experiments and significance determine by a t-test).

1. Fabian MA, Biggs WH, 3rd, Treiber DK, et al. A small molecule-kinase interaction map for clinical kinase inhibitors. Nat Biotechnol 2005; 23: 329-36.
2. Karaman MW, Herrgard S, Treiber DK, et al. A quantitative analysis of kinase inhibitor selectivity. Nat Biotechnol 2008; 26: 127-32.
3. Chen GC, T. D.; Fisher, B.; He, X.; Li, K.; Li, Z.; McGee, L. R.; Pattaropong, V.; Faulder, P.; Seganish, J. L.; Shin, Y. Alkynyl alcohols as kinase inhibitors and their preparation, pharmaceutical compositions and use in the treatment of inflammation and inflammatory disorders. WO2009158011A1. 2009.
